# Editorial

**Published:** 1867-11

**Authors:** 


### Editorial.

---

PROSTITUTION AND SYPHILIS.—In the October number of the EXAMINER we published a letter from Prof. E. ANDREWS, containing the most complete and reliable statistical information concerning these important topics, that we have seen from any source. As the attentive reader will have seen, those facts show conclusively that the attempts perseveringly made in Europe, to limit the spread of syphilis by subjecting prostitutes to legal licenses and inspections, are unqualified failures. And the same results will follow all future laws of the same character, whether in Europe or America, so long as they apply only to *female* prostitutes. Of what avail is it to subject the female to an examination once a-week, while, from the very nature of the case, she may have a poisonous chancre developed the next day after she has received her certificate of health, and inoculate every one with whom she has connexion during the whole week? Of what avail is it to send the diseased female to a prison hospital for cure, while the *male* libertine is allowed to stalk abroad unmolested, his genitals reeking with disease, ready to inoculate the female again the first night after her liberation from the hospital?

When such laws can be enacted, and *rigidly enforced*, as will compel, not only every female, but every male also, proposing to engage in illicit sexual intercourse, to submit to the same registration and inspection, and the same hospital discipline, then some progress may be made in limiting the spread of disease. When a just public sentiment will as rigidly exclude, from all respectable society, every *man*, whether old or young, rich or poor, who is known to indulge in illicit sexual intercourse, as the present public sentiment does the female, then there will be little necessity for statute laws on the subject.

---

INTERNATIONAL CONGRESS.—In the present number of the EXAMINER will be found two letters from Prof. ANDREWS, giving a very interesting account of the doings of the recent International Congress in Paris. From the tenor of all the correspondence we have seen, it is evident that a large proportion of those who attended were disappointed at the character of the proceedings.

---

THE LECTURE SEASON.—The two medical schools in this city are again in active operation, with good classes in attendance on both. The Rush Medical College Faculty made a formal opening and dedication of their new building on the evening of October 2d. The President, Prof. BLANEY, gave a history of the college. Mayor RICE made a congratulatory speech, in which he unwittingly hit the Faculty a blow directly on the head, by congratulating the audience that the capacious new building was the result of "*no joint-stock* enterprise." And

Prof. Gunn delivered the regular introductory lecture. Making due allowance for the puffing and blowing about the "largest and best college building in the *world*," we believe the exercises were satisfactory to all parties. We understand that the number of students in attendance is nearly the same as last year. We fear it will not take many years to satisfy those who boast so much about the "largest and best college building in the world," that something besides brick and mortar is required to constitute a good school of medicine; and that the magnificent spectacle of 200 or 250 students in lecture-rooms with capacity for 600 or 700, will hardly offset the annual drain occasioned by the interest on $70,000 of indebtedness.

The Chicago Medical College, in its neat and admirably arranged, though unpretentious, building, free from pecuniary incumbrances, held its opening exercises on the evening of Oct. 7th. After prayer by the chaplain, and some remarks on the peculiarities of the college organization, by the President of the Faculty, the introductory lecture was delivered by Prof. Nelson. The hall was filled with a good audience, and the lecture was highly appropriate to the occasion. The number of students in attendance is greater than at the commencement of any previous collegiate year. The clinical courses in the several hospitals are also well attended by students eager for observation and instruction directly at the bedside of the patient.

---

American Journal of the Medical Sciences.—The October number of this old Quarterly is promptly on our table. We need hardly remind our readers that this is one of the best medical periodicals in this or any other country.

---

The Medical Gazette.—This is the title of a new weekly journal, published by A. Simpson & Co., New York, and edited by L. M. Yale, M.D. It is a regular medical newspaper, issued every Saturday. Price, $2.00 per annum.

---

L'Evenement Medical.—This is the title of a new weekly medical journal, published in Paris, and edited by Professor Piorry. It is issued in ordinary newspaper form, every Saturday, at 5 francs per annum.

A New Process for Preparing Anatomical Specimens.—Dr. Brunetti, of Padua, who received a gold medal at the Paris Exposition, has generously communicated to the international Medical Congress the following particulars of his valuable invention. The process comprises four several operations, *viz.*, 1, the washing of the piece to be preserved; 2, the *degraissage*, or eating away of the fatty matter; 3, the tanning; and 4, the desiccation.

1. To wash the piece, M. Brunetti passes a current of pure water through the bloodvessels and the various excretory canals, and then he washes the water out by a current of alcohol.

2. For destroying the fat, he follows the alcohol with ether, which he pushes, of course, through the same bloodvessels and excretory ducts; this part of the operation lasts some hours. The ether penetrates the interstices of the flesh, and dissolves all the fat. The piece, at this point of the process, may be preserved any length of time desired, plunging in ether, before proceeding to the final operations.

3. For the tanning process, M. Brunetti dissolves tannin in boiling distilled water, and then, after washing the ether out of the vessels with distilled water, he throws this solution in.

4. For the drying process, M. Brunetti places the pieces in a vase with a double bottom, filled with boiling water, and he fills the places of the preceding liquids with warm, dry air. By the aid of a reservoir, in which air is compressed to about two atmospheres, and which communicates by a stop-cock and a system of tubes, first to a vase containing chloride of calcium, then with another heated, then with the vessels and excretory ducts of the anatomical piece in course of preparation, he establishes a gaseous current which expels, in a very little time, all the fluids. The operation is now finished.

The piece remains supple, light, preserves its size, its normal relations, its solid elements, for there are no longer any fluids in it. It may be handled without fear, and will last indefinitely. The discovery is a magnificent one, and the sooner medical schools are provided with full cabinets of natural and pathological pieces the better.—*Medical and Surgical Reporter.*

---

Liebig's Artificial Milk is in imitation, as close as chemistry can make, of the natural food of the human infant. It is prepared as follows: Half an ounce of wheat flour is boiled to a paste in five ounces of skimmed milk. To this is added immediately a mixture of one-half ounce of bruised malt, one ounce of water, and three grammes of a solution of two parts of bicarbonate of potassa in eleven parts of water. The whole is then kept warm by standing within an envelope of tepid water until it is no longer pastry, but of a creamy consistence. After fifteen or twenty minutes it is put on a fire for a few seconds only, and then strained through a fine hair sieve. It should be allowed to stand long enough to deposit some fibrous matter before it is given to the child or invalid.—*Phil. Med. Reporter.*

---

Heat as a Resuscitating Agent.—Dr. J. G. Richardson, in the *Am. Jour.*, urges from his observation and experiments the importance of direct Heat as an agent in the restoration of still-born infants, in cases of asphyxia from drowning, hanging, or the inhalation of noxious gases, especially the vapor of chloroform. He advises not merely *warming* the surface but artificially warming the blood within the limbs of persons apparently dead, and then propelling by frictions towards the heart as rapidly as possible. The heart's pulsations in many instances really take place, feebly, for a considerable time after being undiscoverable by external observation. The heat should approximate *roasting* as nearly as may be without positive destruction of the tissues. Mere vesication he thinks ought not to be considered more than a minor evil in comparison with the cessation of life.

---

Citrate of Soda in Glucosuria.—A French writer, quoted in *Ranking's Abstract*, recommends citrate of soda in daily doses of a-half drachm to a drachm as a remedy in diabetes. It has been shown by analysis, he says, that sugar disappears from the urine when this salt is used with the food instead of common salt.—*Pacific Med. & Surg. Journal.*

---

Phosphorus Pills.—The formula of Dr. Radcliffe is as follows:—Phosphorus 6 grains, suet 600 grains. Melt the suet in a stoppered bottle capable of holding twice the quantity. Put in the phosphorus, and shake until the mixture becomes solid. Roll into three-grain pills, and cover with gelatine. Each pill will contain the thirty-third of a grain of phosphorus.—*Ibid.*

---

Death from Swallowing Two Ounces of Chloroform.—Dr. D. W. Stormont, of Topeka, Kansas, reports (*Leavenworth Medical Herald*) a case of suicide by the internal administration of chloroform. The patient was 26 years of age, and in good health at the time. He swallowed two ounces of undiluted chloroform at a single draught. In three minutes after he had laid himself composedly down, he could with difficulty be aroused from the stupor into which he was rapidly sinking; and though he could not speak, he indicated that he had severe pain in the region of the stomach. In five minutes, he was entirely unconscious and breathing stertorously. He died, in just one hour after taking the draught. Medical assistance, from some cause, did not arrive until a few minutes before he died, and nothing was done to counteract the effects of the poison. At the *post mortem* examination, the surface generally was livid; the face, neck, chest, and nails very much so. Bloody froth was issuing from the mouth and nostrils. On opening the chest, both lungs were found to be dark externally, and fully distended. They were uniformly congested with dark, liquid blood, and the posterior portions were perfectly engorged with it. Both sides of the heart were nearly full of black, uncoagulated blood; the liver and spleen both normal externally, but somewhat softened, and filled with dark, liquid blood. The œsophagus was congested. The stomach, at the cardiac end, and along the greater curvature, and half way up each side, was discolored externally, dotted over with ecchymosed-looking patches, giving it a mottled appearance. It contained two or three ounces of a light-colored liquid, which had a slight odor of chloroform. At the cardiac end, internally, and along the bottom, nearly to the pyloric end, the mucous membrane was of a dark-red color, softened, and easily peeled off with the thumb-nail. Up the sides, it was of a brighter red, speckled appearance, and not softened. The intestines were healthy. Circumstances prevented the extension of the examination, which is much to be regretted. The reporter of the case closes with the following remarks:—"Recoveries are recorded from drinking two ounces, or even more, of chloroform, but active measures were used—as the stomach-pump, emetics, stimulants, internal and external artificial respiration, galvanism, etc. As an internal stimulant, the spirits of ammonia, or the carbonate of ammonia, is the best. Very dangerous symptoms have been produced by half an ounce, and death has been caused by one ounce. Dr. Stillé says:—'When death has been produced by the internal use of chloroform, its local irritant action has evidently been the chief cause of the fatal result.' In this case, death followed too soon to have been produced in this way. It was more probably caused by the action of the poison on the blood and the cerebro-spinal system, as in prolonged inhalation of the vapor. A peculiarity of this case, is the shortness of the time between taking the chloroform and death, as compared with other fatal cases reported."

Mortality Report for the Month of September:—

Dr. Rauch submitted the following mortality report for the month of September, 1867:—

CAUSES OF DEATH.

| | | | |
|---|---|---|---|
| Abscess, pelvic | 1 | Fever, Scarlet, | 3 |
| Abscess, psoas | 1 | Fever, Typhoid, | 18 |
| Accidents, | 12 | Fever, Typhus, | 4 |
| Œdema-glottidis, | 1 | Gangrene of foot, | 1 |
| Anæmia, | 1 | Gangrene of foot and leg, | 1 |
| Angina, | 1 | Gastritis, | 3 |
| Apoplexy, | 3 | Gastritis, chronic, | 1 |
| Atrophy, | 1 | Hæmoptysis, | 2 |
| Birth, premature | 12 | Hæmoptysis, with phthisis, | 1 |
| " still | 22 | Heart Disease, | 8 |
| Bowels, inflammation of | 6 | Heart, valvular disease of, | 2 |
| Bowels, ulceration of | 1 | Hepatitis, chronic, | 1 |
| Brain Disease, | 1 | Hernia, strangulated | 1 |
| " Congestion of, | 4 | Hydrocephalus, | 3 |
| " Dropsy, | 1 | Inanition, | 5 |
| " Inflammation of, | 5 | Laryngitis, | 1 |
| " Pressure of occipital bone, | 1 | Liver, atrophy of | 1 |
| Bronchitis, | 1 | Liver, hypertrophy | 1 |
| Cancer of stomach, | 2 | Lungs, congestion of | 1 |
| " " encephaloid of mesentry | 1 | Measles, | 10 |
| " " uterus, | 1 | Meningitis, | 6 |
| Cerebritis, | 1 | Meningitis, Cerebral, | 2 |
| Cholera-Morbus, | 2 | Meningitis, Cerebro-Spinal, | 3 |
| Cholera Infantum, | 82 | Meningitis, Tubercular | 2 |
| Cholera Infantum and teething, | 2 | Mouth, excessive morbid growth of | 1 |
| Convulsions, | 25 | Œsophagus, inflammation of | 1 |
| Convulsions following measles, | 1 | Ovarian, disease of | 1 |
| Croup, | 1 | Old Age, | 3 |
| Croup, pseudo-membramus, | 1 | Peritonitis, | 2 |
| Cynanche tonsellaris, | 1 | Pharyngitis, | 1 |
| Debility, | 15 | Pneumonia, | 7 |
| Debility from insanity, | 1 | Pneumonia, chronic, | 1 |
| Delirium Tremens, | 1 | Phthisis Pulmonalis, | 24 |
| Diphtheria, | 5 | Phthisis, Bronchial, | 1 |
| Diarrhœa, | 36 | Potts' disease, | 1 |
| Diarrhœa, chronic, | 7 | Small-Pox, | 13 |
| Diarrhœa and teething, | 2 | Suicide by pistol wound, | 2 |
| Dropsy, | 1 | Tabes Mesenterica, | 26 |
| Dropsy, after intermittent fever, | 1 | Tabes, Mesenterica, with thrush, | 1 |
| Dysentery, | 19 | Tetanus, Traumatic, | 1 |
| Dysentry, following measles, | 1 | Teething, | 20 |
| Encephalitis, | 3 | Teething and Diarrhœa, | 2 |
| Encephalo-meningitis, | 1 | Teething and Whooping-Cough, | 1 |
| Enteritis, | 3 | Throat, Canker sore, | 1 |
| Epilepsy, | 1 | Throat, malformation of, | 1 |
| Erysipelas, gangrenus, | 1 | Urine, suppression of, | 1 |
| Fever, Congestive | 4 | Whooping-Cough, | 6 |
| Fever, Intermittent, | 1 | Unknown, | 4 |
| Fever, Puerperal, | 4 | | |

Total, 507

COMPARISON.

Total deaths for the month of September, 1867, ....................507
Total deaths for the month of September, 1666, ....................759

Decrease, ....................252

AGES OF THE DECEASED.—Under 5 years, 338; over 5 and under 10 years, 14; over 10 and under 20, 15; over 20 and under 30, 27; over 30 and under 40, 23; over 40 and under 50, 16; over 50 and under 60, 18; over 60 and under 70, 10; over 70 and under 80, 5; over 80 and under 90, 3; still born and premature, 34; unknown, 4. Total, 507.

NATIVITIES.

| | | | | | |
|---|---|---|---|---|---|
| Chicago, | 311 | Germany, | 42 | Poland, | 1 |
| Other parts U. S., | 75 | Holland, | 3 | Sweden, | 4 |
| Bohemia, | 5 | Hanover, | 1 | Scotland, | 2 |
| Canada, | 5 | Ireland, | 35 | Switzerland, | 3 |
| Denmark, | 3 | Norway, | 8 | Unknown, | 1 |
| England, | 5 | Prussia, | 3 | Total, | 507 |

SEXES.

Males, ....268 | Females, ....239 | Total, ....507

MARRIED AND SINGLE.

Single, ....428 | Married, .... 79 | Total, ....507
Colored, ....3 | White, ....504 | Total, ....507

DEATHS IN EACH WARD.

The following table gives the average of deaths in each ward, on the basis of the population of 1866:

| Ward. | Mortality. | Pop. in 1866. | One death in | Ward. | Mortality. | Pop. in 1866. | One death in |
|---|---|---|---|---|---|---|---|
| 1 | 1 | 9,668 | 9,668 | 14 | 34 | 12,108 | 356 4-34 |
| 2 | 10 | 12,985 | 1,298 1-2 | 15 | 50 | 15,766 | 315 16-50 |
| 3 | 15 | 15,738 | 1,049 3-15 | 16 | 31 | 14,912 | 481 1-31 |
| 4 | 22 | 10,884 | 494 16-24 | County hos., | 10 | | |
| 5 | 34 | 9,610 | 223 28-24 | Canal, | 1 | | |
| 6 | 34 | 10,580 | 311 6-34 | Bridewell, | 1 | | |
| 7 | 69 | 18,755 | 271 56-69 | Home of the Friendl's, | 5 | | |
| 8 | 40 | 10,429 | 260 29-40 | | | | |
| 9 | 28 | 13,940 | 497 24-28 | Marine hos., | 1 | | |
| 10 | 12 | 11,416 | 951 1-3 | Mercy hos., | 2 | | |
| 11 | 35 | 12,924 | 369 9 35 | Orph. asy., | 3 | | |
| 12 | 43 | 12,695 | 295 10-43 | St. Luke hos. | 1 | | |
| 13 | 24 | 8,188 | 341 1-6 | Lake Mich., | 1 | | |

Total, ....................507

---

DENSITY OF OZONE.—M. Soret has proved the density of ozone to be one and a-half times that of oxygen, by the test of diffusion, the relative velocities of which correspond to the theoretical calculation on the assumption of the above proportion; as well as by comparisons of volume, in which it is found that by converting ozone into oxygen, its volume is increased one-half.

Money Receipts to October 22d.—Drs. D. J. Hussey, Cherry Valley, Ill., $3; T. T. Ellis, Chicago, 3; Wm. Law, Shullsburgh, Wis., 3.75; H. N. Hurlbut, Chicago, 2; A. C. Simonton, Logan, Ind., 6; A. S. Stewart, Pawnee City, Neb., 2; J. Woodworth, Marengo, Ill., 3.

---

To Physicians.—By request, Prof. Horatio R. Storer will deliver his second private course of twelve Lectures upon the Treatment of the Surgical Diseases of Women, during the first fortnight of December, at his rooms in Boston. Fee $50, and Diploma required to be shown.

Certificates of attendance upon the course just completed have been issued to the following gentlemen: Dr. C. M. Carleton, Norwich, Ct.; Daniel Mann, Pelham, N.H.; G. E. Bullard, Blackstone, Mass.; J. A. McDonough, Boston, Mass.; M. C. Talbott, Warren, Pa.; H. Gerould, Erie, Pa.; E. F. Upham, West Randolph, Vt.; W. L. Wells, Howall, Mich.; and W. A. I. Case, Hamilton, C.W.

*Hotel Pelham, Boston, 1st July, 1867.*

